# Expanding Family Health History to Include Family Medication History

**DOI:** 10.3390/jpm13030410

**Published:** 2023-02-25

**Authors:** Susanne B. Haga, Lori A. Orlando

**Affiliations:** Department of Medicine, School of Medicine, Duke University, Durham, NC 27708, USA

**Keywords:** family health history, medication history, pharmacogenetic testing

## Abstract

The collection of family health history (FHH) is an essential component of clinical practice and an important piece of data for patient risk assessment. However, family history data have generally been limited to diseases and have not included medication history. Family history was a key component of early pharmacogenetic research, confirming the role of genes in drug response. With the substantial number of known pharmacogenes, many affecting response to commonly prescribed medications, and the availability of clinical pharmacogenetic (PGx) tests and guidelines for interpretation, the collection of family medication history can inform testing decisions. This paper explores the roots of family-based pharmacogenetic studies to confirm the role of genes in these complex phenotypes and the benefits and challenges of collecting family medication history as part of family health history intake.

## 1. Introduction

The use of family health history (FHH) data has experienced a resurgence of sorts with the development of electronic medical records, clinical decision supports, and patient portals facilitating the collection and utilization of this valuable information [1]. Alone or in combination with genetic or genomic technologies, FHH is an important tool in health risk assessment for both genetic (single gene) diseases and common, complex diseases [2,3,4,5,6,7]. Perhaps overlooked due to the predominant focus on disease risk assessment, family history can also inform therapeutic decisions. Indeed, FHH played a critical role in defining the genetic etiologies of response to medications in the early days of pharmacogenetic (PGx) research. Thus, with the substantial number of known pharmacogenes, many affecting response to commonly prescribed medications, the inclusion of family medication history in FHH can inform PGx testing and treatment decisions. This paper explores the roots of family-based PGx studies to identify genetic causes of drug response and considers the benefits and challenges of collecting family medication history as part of family health history intake.

## 2. A Quick History of Family Health History & PGx 

As today’s FHH intake primarily focuses on diseases and risk assessment, other health outcomes such as drug response have not received the same recognition but may benefit from consideration within the family health history intake process. Historically, the study of families was instrumental to deciphering the role of genes in drug response, which, in some instances, informed treatment decisions. While adverse responses to foods and medicines have been documented for centuries, the recognition of the importance of FHH was demonstrated by Sir Archibald Garrod and his study of families with inherited biochemical disorders. His prescient work in biochemical genetics also recognized individuals’ different responses to pharmacological treatment [8]. 

With discoveries of Mendelian-like adverse drug responses in the mid-1900s, FHH continued to be a key part of studies to ascertain the role of genetic factors. For example, the early use of isoniazid in tuberculosis patients demonstrated a bimodal pattern of metabolism (Biehl 1957, 1958). Multiple family studies soon confirmed the genetic cause of the slow/rapid rates of isoniazid metabolism. Knight et al. (1959) published their analysis of 91 individuals from 20 families, concluding an autosomal recessive inheritance pattern [9]. 

At about the same time, other researchers were studying the enzyme pseudocholinesterase. In 1956, Lehmann and Ryan [10] studied five families with prolonged apnea following suxamethonium treatment and demonstrated differences in pseudocholinesterase activity. The final sentence of their conclusion reads as follows: “It now seems to us that not only should every patient who has a prolonged apnea after suxamethonium be examined for a lowered pseudocholinesterase level, but that his relations should be investigated as well.” Following Lehman and Ryan’s family studies, Kalow and Staron [11] shortly thereafter published their work confirming heterozygous and homozygous family members with respect to enzyme activity levels (Kalow reflected that his desire for more data from families resulted in his team getting scooped [12]). In 1960, Evans et al. [13] analyzed 267 individuals from 53 families and confirmed the recessive mode of inheritance of pseudocholinesterase levels. 

In yet another example in 1960, a case report was published of a young male patient in Australia who was scheduled to undergo surgery for a compound fracture of the tibia and fibula [14]. The patient expressed concern about the use of general anesthesia due to several deaths of family members following surgery. An extensive analysis of the proband’s family history confirmed the use of ethyl chloride and ether in 10 family members in two generations who had died following surgery [15]. A dominant mode of inheritance was predicted with the possibility of incomplete penetrance. In 1990, the gene responsible for malignant hyperthermia was discovered, though family history remains a key step in the discovery of new variants and a key predictor of malignant hyperthermia risk [16,17,18,19]. Similarly, the genetic role of adverse responses to anti-depressant treatments was demonstrated through family-based studies [20,21,22]; and in more recent years, twin studies have confirmed the genetic contribution to most drug responses [23,24].

Today, the clinical use of whole genome and whole exome sequencing continues to expand for disease diagnosis and risk assessment, yet its role and value, alone or in combination with FHH, has not been unequivocally demonstrated. In a study of 1750 healthy Singaporeans that had both a high-quality FHH and whole genome sequencing, FHH information defined the cancer-related clinical care strategy in 98% of participants [25]. Specifically, combined whole genome and FHH identified an increased risk in only 1.5% of participants (no increased risk observed in 89.5% of participants). In this setting, the FHH overwhelmingly determined risk management strategies, with only about 2% of participants identified at increased risk based on genome sequencing not predicted by FHH. Another study of sequencing of patients at risk of autosomal dominant polycystic kidney disease reported a higher rate of a positive sequencing result in those with a family history (91.3%) to those without (50.6%) [26]. Patients undergoing whole exome or genome clinical sequencing currently have the option to receive secondary results for only two pharmacogenes (RYR1 and CACNA1S—both related to malignant hyperthermia) [27]. 

## 3. Expanding Family Health History to Include Family Medication History

Despite the well-documented role of inheritance of adverse events for select drugs, family medication history is not typically included as part of the standard FHH intake. In 2016, Smith et al. suggested that family medication history be collected and utilized to inform decisions about PGx testing [28]. Medication histories are often taken by a pharmacist, often as part of medication therapy management, including the patient’s history of and current medication use. However, expansion of FHH to include family medication history regarding drug efficacy and adverse drug reactions from three generations could improve therapeutic clinical decision-making and/or if PGx testing is warranted [28,29,30]. Examples include venous thromboembolism where a family history alters the risk/benefit balance in the decision to prescribe oral contraceptives [31,32], opioid-adverse events where a family history can alter the choice and dose of opioid prescribed [33], and vancomycin-induced drug reactions with eosinophilia and systemic symptoms syndrome [34]. The recent attention and improvement in tools to collect and utilize FHH provides a timely opportunity to include family medication history. 

Despite its potential, establishing broader PGx testing continues to be limited. A family medication history may indicate a need for PGx testing to confirm an increased risk of adverse events or non-response to certain medications. Family history of Stevens-Johnson syndrome [35] can inform decisions to undergo pre-emptive PGx testing prior to initiating anticonvulsant medications. In addition, as with diseases, cascade testing of family members of those identified with a PGx susceptibility can be a useful adjunct to clinical paradigms [36,37]. 

There are some specialties and clinical scenarios in which medication history is typically collected. For example, psychiatrists collect a medication history during a new patient visit to understand their prior drug experiences. This information can then be used to inform future treatment decisions, with the goal of avoiding both ineffective medications and adverse events [38,39]. Other specialties that prioritize a detailed medication history are: obstetrics during the first prenatal visit to ensure teratogenic and other medications dangerous to embryonic and fetal development are withheld; oncology given the significant toxicities of most chemotherapeutic drugs; and both nephrology and hepatology since dysfunction of these organs dramatically alters drug metabolism and excretion. Outside of specific specialties, all physicians that prescribe certain systemic medications with narrow therapeutic indexes and high-risk drug-drug interactions collect a detailed medication history [40]. Additionally, providers may benefit during the collection of a family medication history by enhancing their understanding of family experiences with, or attitudes towards, medications that ultimately may influence patient medication compliance behavior. One study reported that family medication history was associated with pediatric use of supplements [41]. 

## 4. Discussion—Challenges to Clinical Implementation

Some challenges should be considered when expanding FHH to include family medication history. It has been well-documented that FHH is under-utilized for a variety of reasons [42,43,44,45] and thus, suggesting collection of further information may not be feasible. In particular, evidence suggests that FHH is under-reported [46,47,48,49,50] and potentially inaccurate or incomplete [51,52,53], with patient recall, sharing, and communication impacted by multiple factors including unawareness, differences in knowledge of maternal vs. paternal family histories, patient gender, degree of relatives, and cultural factors [54,55,56,57,58,59,60,61,62]. 

Discrepancies between self-reported medication history and prescription history in the medical record have been demonstrated [63,64], highlighting patient unawareness, confusion, and poor recollection [65]. Additionally, patient reporting of adverse events has been reported to be low [66]. Reconstruction of a patient’s medication history from the medical record, if feasible, is likely to be time-consuming and incomplete [67]. Some inconsistencies in patient reporting of current adverse drug responses have also been reported due to difficulty with attribution regarding multiple medications and poor recall [68]. Although it is unclear whether the same challenges will be true for family medication history and knowledge of adverse drug reactions in family members, there are several reasons to believe that it may actually be more difficult. First, medication names can be more difficult to recall than disease names and knowledge of adverse events may not include knowledge of the specific medication. Second, the pace of FDA approval for novel medications and classes of medications has been rapidly increasing over the last 50 years, and many medications available today were not available for earlier family generations. Third, determining whether a symptom is in fact a medication-induced adverse event is often difficult to determine. Lastly, as with diseases, patient recall may be biased toward more serious adverse events or poor outcomes related to lack of efficacy or serious reactions. 

There has been limited evaluation of the possible solutions that could enhance uptake and accuracy of FHH and medication history. For FHH, several digital-based tools have been developed and evaluated to promote FHH collection and analysis [69,70,71,72]. 

For medication history, many digital applications have been developed to improve medication adherence, though the quality and effectiveness are debatable [73]. These same adherence apps could serve as a record of current medications to be shared with a provider. One strategy to improve accuracy is the careful formulation of the intake questions to specifically focus on medications known to be impacted by pharmacogenes or formatted as open-ended questions [74]. Use of a patient-friendly format, such as the patient-reporting version of the UK Yellow Card Scheme, may facilitate accurate reporting of adverse events [75]. Consistent self-reporting of adverse events may identify a drug-metabolizing deficiency or other key protein abnormality that can be confirmed by a PGx test and, therefore, the questions should be included on the intake forms completed for each office visit. Medication-specific questions may be extracted from the many instruments for patient-reported outcome measures (PROMs) developed over the past decade [76,77,78]. Another approach is to combine medication history with FHH such that reporting of disease diagnoses in family members could include questions regarding medications and medication response for that given disease. Patient educational materials will be important to facilitate understanding of what types of information should be reported. While decisions made on incomplete data can lead to treatment errors and poor outcomes, it is better to gather some information than none at all. Well-designed research of these strategies can inform implementation of effective methods.

Lastly, the collection and review of additional information will likely add to the provider’s consultation time per patient, and potentially the need for more healthcare professionals, especially pharmacists and multidisciplinary teams [66,79,80]. Digital tools to facilitate documentation of medication (e.g., medication tracking apps) and adverse events may improve accuracy and timely reporting [81] and avoid inaccuracies associated with patient recall.

## 5. Conclusions

As with any medical condition, predicting response to medications will be impacted by awareness of multiple factors, some yet to be determined. The combination of FHH and medication history, potentially with PGx test results, may yield a better predictor for medication use and improved health outcomes. As new FHH tools and resources continue to be developed and updated, these may facilitate reporting of family medication usage and responses. Thus, it may be worthwhile to consider the inclusion of adding family medication history to routine patient intake processes to identify patients at increased susceptibility for an adverse response. Further research is needed to demonstrate the utility of an enhanced FHH to include medication history and strategies to facilitate accurate and complete patient reporting. Greater attention to patient and family history of medications may indirectly promote awareness, medication adherence, and recognition and reporting of adverse events.

## Data Availability

Not applicable.

## References

[1] Welch B.M., Wiley K., Pflieger L., Achiangia R., Baker K., Hughes-Halbert C., Morrison H., Schiffman J., Doerr M. (2018). Review and Comparison of Electronic Patient-Facing Family Health History Tools. J. Genet. Couns..

[2] Rudichuk L., Vogel K.J., Wang C.H., Helfand B.T., Selkirk C.G. (2017). Urologists’ Current Practices in Screening and Treating Men with a Family History of Prostate Cancer. Urology.

[3] Moonesinghe R., Beckles G.L.A., Liu T., Khoury M.J. (2018). The contribution of family history to the burden of diagnosed diabetes, undiagnosed diabetes, and prediabetes in the United States: Analysis of the National Health and Nutrition Examination Survey, 2009–2014. Genet. Med..

[4] Kim C., Chang H.J., Cho I., Sung J.M., Choi D., Jeong M.H., Jang Y.S. (2013). Impact of family history on the presentation and clinical outcomes of coronary heart disease: Data from the Korea Acute Myocardial Infarction Registry. Korean J. Intern. Med..

[5] Lowery J.T., Ahnen D.J., Schroy P.C., Hampel H., Baxter N., Boland C.R., Burt R.W., Butterly L., Doerr M., Doroshenk M. (2016). Understanding the contribution of family history to colorectal cancer risk and its clinical implications: A state-of-the-science review. Cancer.

[6] Flória-Santos M., Lopes-Júnior L.C., Alvarenga Lde M., Ribeiro M.S., Ferraz V.E., Nascimento L.C., Pereira-da-Silva G. (2016). Self-reported cancer family history is a useful tool for identification of individuals at risk of hereditary cancer predisposition syndrome at primary care centers in middle-income settings: A longitudinal study. Genet. Mol. Biol..

[7] Kumerow M.T., Rodriguez J.L., Dai S., Kolor K., Rotunno M., Peipins L.A. (2022). Prevalence of Americans reporting a family history of cancer indicative of increased cancer risk: Estimates from the 2015 National Health Interview Survey. Prev. Med..

[8] Garrod A.E. (1931). Inborn Factors in Disease: An Essay.

[9] Knight R.A.S.M., Harris H.W. (1959). Genetic factors influencing blood levels in humans. Proceedings of the Veterans Administration 18th Conference of Chemotherapy of Tuberculosis.

[10] Lehman H., Ryan E. (1956). The familial incidence of low pseudocholinesterase level. Lancet.

[11] Kalow W. (1956). Familial Incidence of Low Pseudocholinesterase Level. Lancet.

[12] Jones D.S. (2013). How Personalized Medicine Became Genetic, and Racial: Werner Kalow and the Formations of Pharmacogenetics. J. Hist. Med. Allied Sci..

[13] Evans D.A., Manley K.A., Mc K.V. (1960). Genetic control of isoniazid metabolism in man. Br. Med. J..

[14] Denborough M.A., Lovell R.R.H. (1960). Anaesthetic Deaths in a Family. Lancet.

[15] Denborough M.A., Forster J.F., Lovell R.R., Maplestone P.A., Villiers J.D. (1962). Anaesthetic deaths in a family. Br. J. Anaesth..

[16] Beebe D., Puram V.V., Gajic S., Thyagarajan B., Belani K.G. (2020). Genetics of Malignant Hyperthermia: A Brief Update. J. Anaesthesiol. Clin. Pharmacol..

[17] Quane K.A., Keating K.E., Healy J.M., Manning B.M., Krivosic-Horber R., Krivosic I., Monnier N., Lunardi J., McCarthy T.V. (1994). Mutation screening of the RYR1 gene in malignant hyperthermia: Detection of a novel Tyr to Ser mutation in a pedigree with associated central cores. Genomics.

[18] Matos A.R., Sambuughin N., Rumjanek F.D., Amoedo N.D., Cunha L.B., Zapata-Sudo G., Sudo R.T. (2009). Multigenerational Brazilian family with malignant hyperthermia and a novel mutation in the RYR1 gene. Braz. J. Med. Biol. Res..

[19] Brown R.L., Pollock A.N., Couchman K.G., Hodges M., Hutchinson D.O., Waaka R., Lynch P., McCarthy T.V., Stowell K.M. (2000). A novel ryanodine receptor mutation and genotype-phenotype correlation in a large malignant hyperthermia New Zealand Maori pedigree. Hum. Mol. Genet..

[20] Franchini L., Serretti A., Gasperini M., Smeraldi E. (1998). Familial concordance of fluvoxamine response as a tool for differentiating mood disorder pedigrees. J. Psychiatr. Res..

[21] Pare C.M., Mack J.W. (1971). Differentiation of two genetically specific types of depression by the response to antidepressant drugs. J. Med. Genet..

[22] Pare C.M., Rees L., Sainsbury M.J. (1962). Differentiation of two genetically specific types of depression by the response to anti-depressants. Lancet.

[23] Theisen F.M., Gebhardt S., Haberhausen M., Heinzel-Gutenbrunner M., Wehmeier P.M., Krieg J.C., Kühnau W., Schmidtke J., Remschmidt H., Hebebrand J. (2005). Clozapine-induced weight gain: A study in monozygotic twins and same-sex sib pairs. Psychiatr. Genet..

[24] Gebhardt S., Theisen F.M., Haberhausen M., Heinzel-Gutenbrunner M., Wehmeier P.M., Krieg J.C., Kühnau W., Schmidtke J., Remschmidt H., Hebebrand J. (2010). Body weight gain induced by atypical antipsychotics: An extension of the monozygotic twin and sib pair study. J. Clin. Pharm. Ther..

[25] Bylstra Y., Lim W.K., Kam S., Tham K.W., Wu R.R., Teo J.X., Davila S., Kuan J.L., Chan S.H., Bertin N. (2021). Family history assessment significantly enhances delivery of precision medicine in the genomics era. Genome Med..

[26] Chang A.R., Moore B.S., Luo J.Z., Sartori G., Fang B., Jacobs S., Abdalla Y., Taher M., Carey D.J., Triffo W.J. (2022). Exome Sequencing of a Clinical Population for Autosomal Dominant Polycystic Kidney Disease. JAMA.

[27] Miller D.T., Lee K., Chung W.K., Gordon A.S., Herman G.E., Klein T.E., Stewart D.R., Amendola L.M., Adelman K., Bale S.J. (2021). ACMG SF v3.0 list for reporting of secondary findings in clinical exome and genome sequencing: A policy statement of the American College of Medical Genetics and Genomics (ACMG). Genet. Med..

[28] Smith T.R., Kearney E., Hulick P.J., Kisor D.F. (2016). History repeats itself: The family medication history and pharmacogenomics. Pharmacogenomics.

[29] Owusu-Obeng A., Weitzel K.W., Hatton R.C., Staley B.J., Ashton J., Cooper-Dehoff R.M., Johnson J.A. (2014). Emerging roles for pharmacists in clinical implementation of pharmacogenomics. Pharmacotherapy.

[30] Wong D.Y., Fogel B.L. (2021). Acute pharmacogenetic dystonic reactions in a family with the CYP2D6 *41 allele: A case report. J. Med. Case Rep..

[31] van Vlijmen E.F., Wiewel-Verschueren S., Monster T.B., Meijer K. (2016). Combined oral contraceptives, thrombophilia and the risk of venous thromboembolism: A systematic review and meta-analysis. J. Thromb. Haemost..

[32] Bezemer I.D., van der Meer F.J.M., Eikenboom J.C.J., Rosendaal F.R., Doggen C.J.M. (2009). The Value of Family History as a Risk Indicator for Venous Thrombosis. Arch. Intern. Med..

[33] Khan N.F., Bateman B.T., Landon J.E., Gagne J.J. (2019). Association of Opioid Overdose With Opioid Prescriptions to Family Members. JAMA Intern. Med..

[34] DeMaio A., Carlock S., Winterfield L.S. (2021). Vancomycin-induced drug reactions with eosinophilia and systemic symptoms syndrome in a patient with positive family history. Dermatol. Online J..

[35] Tangamornsuksan W., Chanprasert S., Nadee P., Rungruang S., Meesilsat N., Ueta M., Lohitnavy M. (2020). HLA genotypes and cold medicine-induced Stevens-Johnson syndrome/toxic epidermal necrolysis with severe ocular complications: A systematic review and meta-analysis. Sci. Rep..

[36] Roosan D., Hwang A., Roosan M.R. (2021). Pharmacogenomics cascade testing (PhaCT): A novel approach for preemptive pharmacogenomics testing to optimize medication therapy. Pharm. J..

[37] Qiumei G., Zhengju C., Xuefei H., Qu C. (2022). Association of HLA-C*01:02 with methazolamide-induced toxic epidermal necrolysis. BMJ Case Rep..

[38] Haro J.M., Lamy F.X., Jönsson B., Knapp M., Brignone M., Caillou H., Chalem Y., Hammer-Helmich L., Rive B., Saragoussi D. (2018). Characteristics of patients with depression initiating or switching antidepressant treatment: Baseline analyses of the PERFORM cohort study. BMC Psychiatry.

[39] American Psychological Association APA Clinical Practice Guideline for the Treatment of Depression Across Three Age Cohorts. https://www.apa.org/depression-guideline/guideline.pdf.

[40] Ricardo J.W., Lipner S.R. (2020). Safety of current therapies for onychomycosis. Expert Opin. Drug Saf..

[41] Kubota M., Mori N., Hamada S., Nagai A., Seto S., Suehiro Y., Kusunoki T., Wakazono Y., Kiyomasu T. (2012). Association of age and family history with supplement use in pediatric patients with allergy. Nutr. Res..

[42] Hussein N., Malik T.F.A., Salim H., Samad A., Qureshi N., Ng C.J. (2020). Is family history still underutilised? Exploring the views and experiences of primary care doctors in Malaysia. J. Community Genet..

[43] Ahmed S., Hayward J., Ahmed M. (2016). Primary care professionals’ perceptions of using a short family history questionnaire. Fam. Pract..

[44] Armel S.R., McCuaig J., Gojska N., Demsky R., Maganti M., Murphy J., Rosen B. (2015). All in the Family: Barriers and Motivators to the Use of Cancer Family History Questionnaires and the Impact on Attendance Rates. J. Genet. Couns..

[45] Ginsburg G.S., Wu R.R., Orlando L.A. (2019). Family health history: Underused for actionable risk assessment. Lancet.

[46] Anton-Culver H., Kurosaki T., Taylor T.H., Gildea M., Brunner D., Bringman D. (1996). Validation of family history of breast cancer and identification of the BRCA1 and other syndromes using a population-based cancer registry. Genet. Epidemiol..

[47] Mitchell R.J., Brewster D., Campbell H., Porteous M.E., Wyllie A.H., Bird C.C., Dunlop M.G. (2004). Accuracy of reporting of family history of colorectal cancer. Gut.

[48] Ziogas A., Anton-Culver H. (2003). Validation of family history data in cancer family registries. Am. J. Prev. Med..

[49] Elbaz A., McDonnell S.K., Maraganore D.M., Strain K.J., Schaid D.J., Bower J.H., Ahlskog J.E., Rocca W.A. (2003). Validity of family history data on PD: Evidence for a family information bias. Neurology.

[50] Ozanne E.M., O’Connell A., Bouzan C., Bosinoff P., Rourke T., Dowd D., Drohan B., Millham F., Griffin P., Halpern E.F. (2012). Bias in the reporting of family history: Implications for clinical care. J. Genet. Couns..

[51] Janssens A.C., Henneman L., Detmar S.B., Khoury M.J., Steyerberg E.W., Eijkemans M.J., Mushkudiani N., Oostra B.A., van Duijn C.M., Mackenbach J.P. (2012). Accuracy of self-reported family history is strongly influenced by the accuracy of self-reported personal health status of relatives. J. Clin. Epidemiol..

[52] Hastrup J.L. (1985). Inaccuracy of family health information: Implications for prevention. Health Psychol..

[53] Chavez-Yenter D., Goodman M.S., Chen Y., Chu X., Bradshaw R.L., Lorenz Chambers R., Chan P.A., Daly B.M., Flynn M., Gammon A. (2022). Association of Disparities in Family History and Family Cancer History in the Electronic Health Record With Sex, Race, Hispanic or Latino Ethnicity, and Language Preference in 2 Large US Health Care Systems. JAMA Netw. Open.

[54] Lin J., Marcum C.S., Myers M.F., Koehly L.M. (2018). Racial differences in family health history knowledge of type 2 diabetes: Exploring the role of interpersonal mechanisms. Transl. Behav. Med..

[55] Lin J., Marcum C.S., Wilkinson A.V., Koehly L.M. (2018). Developing Shared Appraisals of Diabetes Risk through Family Health History Feedback: The Case of Mexican-Heritage Families. Ann. Behav. Med..

[56] Sanghavi K., Moses I., Moses D., Gordon A., Chyr L., Bodurtha J. (2019). Family health history and genetic services-the East Baltimore community stakeholder interview project. J. Community Genet..

[57] Roberts M.C., Krakow M., Wheldon C.W., Silver M.I. (2019). Differences in Family Health History Knowledge among Bisexual and Lesbian Women. LGBT Health.

[58] Rositch A.F., Atnafou R., Krakow M., D’Souza G. (2019). A Community-Based Qualitative Assessment of Knowledge, Barriers, and Promoters of Communicating about Family Cancer History among African-Americans. Health Commun..

[59] Krakow M., Rising C.J., Trivedi N., Yoon D.C., Vanderpool R.C. (2020). Prevalence and Correlates of Family Cancer History Knowledge and Communication among US Adults. Prev. Chronic Dis..

[60] Tehranifar P., Wu H.C., Shriver T., Cloud A.J., Terry M.B. (2015). Validation of family cancer history data in high-risk families: The influence of cancer site, ethnicity, kinship degree, and multiple family reporters. Am. J. Epidemiol..

[61] Sieverding M., Arbogast A.L., Zintel S., von Wagner C. (2020). Gender differences in self-reported family history of cancer: A review and secondary data analysis. Cancer Med..

[62] Orom H., Coté M.L., González H.M., Underwood W., Schwartz A.G. (2008). Family history of cancer: Is it an accurate indicator of cancer risk in the immigrant population?. Cancer.

[63] Choi S.J., Storey R., Parikh S.V., Bostwick J.R. (2019). The Impact of Completing Medication Reconciliation and Depression Treatment History in an Outpatient Depression Clinic. Psychopharmacol. Bull.

[64] Lehnbom E.C., Stewart M.J., Manias E., Westbrook J.I. (2014). Impact of medication reconciliation and review on clinical outcomes. Ann. Pharmacother..

[65] Pogue Y.Z. (2023). Tracking the Medication History of Persons with Serious Mental Disorders. Psychiatr. Serv..

[66] Buchet-Poyau K., Occelli P., Touzet S., Langlois-Jacques C., Figon S., Dubois J.P., Duclos A., Chanelière M., Colin C., Rabilloud M. (2021). Improving patient self-reporting of antihypertensive adverse drug events in primary care: A stepped wedge cluster randomised trial. BMC Fam. Pract..

[67] Iglesias J.E., Rocks K., Jahanshad N., Frias-Martinez E., Andrada L.P., Bui A.A. (2009). Tracking medication information across medical records. AMIA Annu. Symp. Proc..

[68] Arnott J., Hesselgreaves H., Nunn A.J., Peak M., Pirmohamed M., Smyth R.L., Turner M.A., Young B. (2012). Enhancing communication about paediatric medicines: Lessons from a qualitative study of parents’ experiences of their child’s suspected adverse drug reaction. PLoS One.

[69] Qureshi N., Carroll J.C., Wilson B., Santaguida P., Allanson J., Brouwers M., Raina P. (2009). The current state of cancer family history collection tools in primary care: A systematic review. Genet. Med..

[70] Miroševič Š., Klemenc-Ketiš Z., Peterlin B. (2022). Family history tools for primary care: A systematic review. Eur. J. Gen. Pract..

[71] Goldstein K.M., Fisher D.A., Wu R.R., Orlando L.A., Coffman C.J., Grubber J.M., Rakhra-Burris T., Wang V., Scheuner M.T., Sperber N. (2019). An electronic family health history tool to identify and manage patients at increased risk for colorectal cancer: Protocol for a randomized controlled trial. Trials.

[72] Le A., Valice E., Kobelka C., Janes K., Hoodfar E., Powell C.B. (2021). Electronic Family History Screening Tool for Detection of Inherited Cancer Risk: A Prospective Pilot Study. Am. J. Med. Qual..

[73] Backes C., Moyano C., Rimaud C., Bienvenu C., Schneider M.P. (2021). Digital Medication Adherence Support: Could Healthcare Providers Recommend Mobile Health Apps?. Front. Med. Technol..

[74] King A., Daniels J., Lim J., Cochrane D.D., Taylor A., Ansermino J.M. (2010). Time to listen: A review of methods to solicit patient reports of adverse events. Qual. Saf. Health Care.

[75] Avery A.J., Anderson C., Bond C.M., Fortnum H., Gifford A., Hannaford P.C., Hazell L., Krska J., Lee A.J., McLernon D.J. (2011). Evaluation of patient reporting of adverse drug reactions to the UK ‘Yellow Card Scheme’: Literature review, descriptive and qualitative analyses, and questionnaire surveys. Health Technol. Assess.

[76] Weldring T., Smith S.M. (2013). Patient-Reported Outcomes (PROs) and Patient-Reported Outcome Measures (PROMs). Health Serv. Insights.

[77] Oude Voshaar M., Terwee C.B., Haverman L., van der Kolk B., Harkes M., van Woerden C.S., van Breda F., Breukink S., de Hoop I., Vermeulen H. (2023). Development of a standard set of PROs and generic PROMs for Dutch medical specialist care : Recommendations from the Outcome-Based Healthcare Program Working Group Generic PROMs. Qual. Life Res..

[78] James K.A., Cadel L., Hitzig S.L., Guilcher S.J.T. (2022). Patient-reported outcome measures for medication-related quality of life: A scoping review. Res. Social. Adm. Pharm..

[79] Benson H., Lucas C., Benrimoj S.I., Williams K.A. (2019). The development of a role description and competency map for pharmacists in an interprofessional care setting. Int. J. Clin. Pharm..

[80] Hazen A., Sloeserwij V., Pouls B., Leendertse A., de Gier H., Bouvy M., de Wit N., Zwart D. (2021). Clinical pharmacists in Dutch general practice: An integrated care model to provide optimal pharmaceutical care. Int. J. Clin. Pharm..

[81] Holch P., Warrington L., Bamforth L.C.A., Keding A., Ziegler L.E., Absolom K., Hector C., Harley C., Johnson O., Hall G. (2017). Development of an integrated electronic platform for patient self-report and management of adverse events during cancer treatment. Ann. Oncol..

